# A rare case of ectopic retrosternal goiter

**DOI:** 10.1002/ccr3.3610

**Published:** 2020-12-14

**Authors:** Shen Leong Oh, Clement L. K. Chia, Oon Cheong Ooi, Vikram Sonawane, Anil D. Rao, Reyaz Singaporewalla

**Affiliations:** ^1^ Department of General Surgery Khoo Teck Puat Hospital Singapore Singapore; ^2^ Department of Cardiac, Thoracic and Vascular Surgery National University Heart Hospital Singapore Singapore; ^3^ Department of Endocrinology Khoo Teck Puat Hospital Singapore Singapore

**Keywords:** ectopic, intrathoracic, retrosternal goiter, thyroid

## Abstract

It is important for the clinician to be familiar with interpreting a variety of radiological modalities that provide vital information that will aid in the preoperative planning, counseling, and subsequent management of patients with retrosternal goiter.

## Graphical abstract

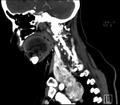

## 1 INTRODUCTION

Retrosternal goiters (RSGs) are commonly defined as one that either descends below the thoracic inlet or has more than 50% of its mass located in the mediastinum. They can be divided into secondary goiters due to extension from a cervical thyroid gland, or primary goiters due to ectopic thyroid tissue in the mediastinum.^1^ The presence of RSG is a relatively common occurrence, documented in 1%–20% of all thyroidectomies. However, findings of an ectopic RSG are a much rarer entity consisting of only 0.2%–1% of all RSGs.^2^

This case reviews the workup and radiological features of a RSG and provides an update on the management of RSG. It is important for the surgeon to be familiar with interpreting the ultrasound and computer tomographic scans as it provides vital information on the anatomical location of the RSG, its relations to the neighboring structures as well as the morphology of the goiter. These will aid in the preoperative planning, counseling, and subsequent management of the patient.

## 2 CASE PRESENTATION

**FIGURE 1:**
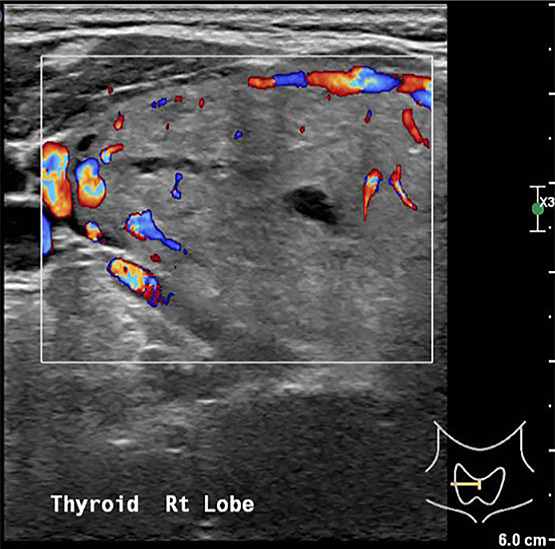
Ultrasound scan of right thyroid lobe

**FIGURE 2:**
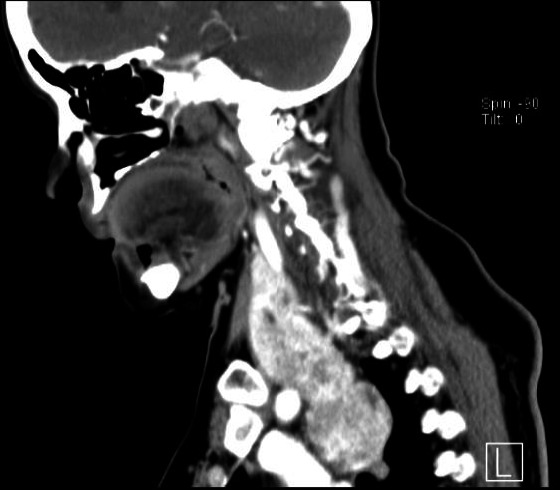
Sagittal section of the CT scan

**FIGURE 3:**
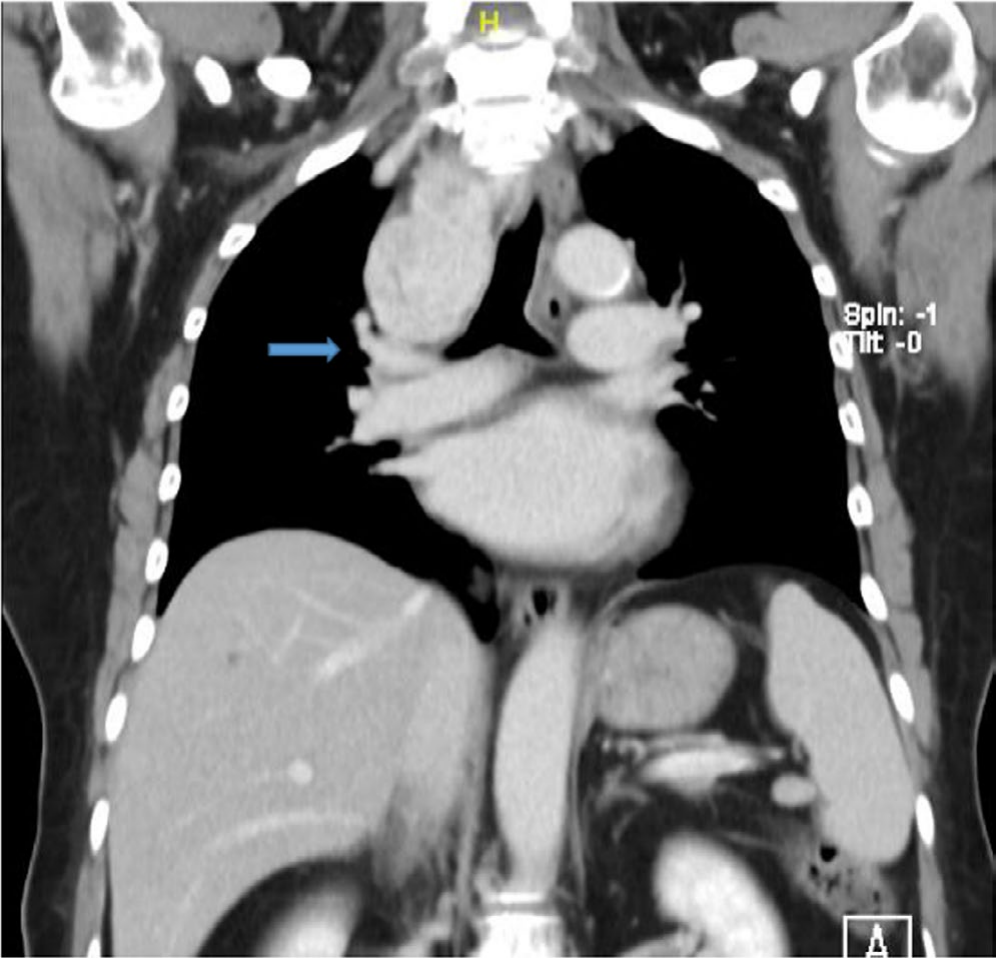
Coronal section of the CT scan

A 69‐year‐old Chinese lady with comorbidities of diabetes and hypertension presented to the emergency department with chest pain and shortness of breath. Physical examination did not reveal any cardiac arrhythmias or lung crepitations. Incidentally, she was found to have a large goiter and it was not possible to get below the goiter on clinical examination. Her trachea was also deviated to the left. There were no associated signs of hyperthyroidism. An acute coronary event was excluded with Electrocardiogram and serial cardiac enzymes. Thyroid function test was also normal. Based on the clinical findings, a thyroid ultrasound (Figure 1) and computer tomographic (CT) scan of neck and thorax (Figures 2 and 3) were performed. What underlying condition does the patient have?

## 3 IMAGE INTERPRETATION

The thyroid ultrasound (Figure 1) shows a well circumscribed isoechoic right thyroid nodule that is predominantly solid replacing the entire right lobe of the thyroid. There is increased peripheral vascularity but no microcalcifications are seen. This thyroid nodule is classified as TIRADS 4a on ultrasound. The CT scan of the neck and thorax confirmed the presence of retrosternal extension of the goiter into the anterior mediastinum (Figure 2). On coronal section (Figure 3), it shows the relationship of the intrathoracic goiter component in close proximity with the right superior pulmonary artery (blue arrow) as well as the presence of a plane separating the two structures.

## 4 DIAGNOSIS

This patient has a large symptomatic goiter with retrosternal extension causing trachea compression with resultant dyspnea.

## 5 CLINICAL PROGRESS

**FIGURE 4:**
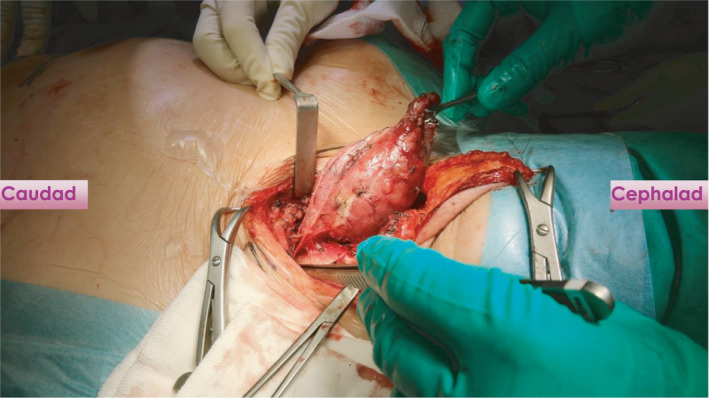
Traction on the cervical goiter being applied cephalad and absence of continuity of the inferior border of the cervical goiter with its intrathoracic component

**FIGURE 5:**
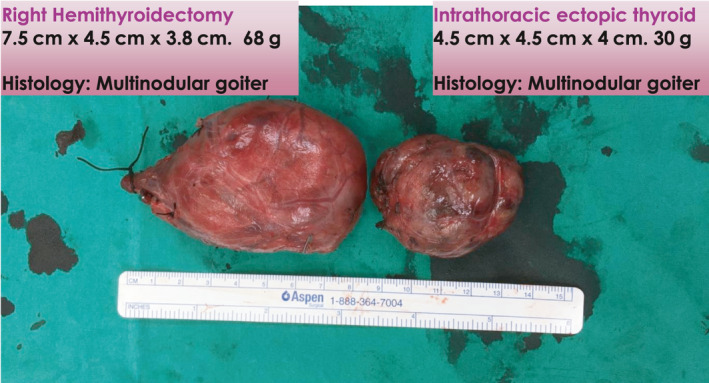
Intraoperative specimens showing the right hemithyroidectomy specimen without continuity with the ectopic intrathoracic component

Fine‐needle aspiration cytology of the right thyroid goiter shows findings compatible with a nodular goiter, Bethesda II. The left lobe of the thyroid had several subcentimeter nodules that were benign looking on ultrasound. Patient was counseled for right hemithyroidectomy with a possibility of video‐assisted thoracoscopic surgery (VATS) in view of retrosternal extension. A conventional right hemithyroidectomy was performed via the cervical approach first with the patient in supine position and neck extended. We perform the lateral trapdoor approach^3^ in our institution and divided the strep muscles to improve access to the thyroid. After mobilizing the thyroid, the recurrent laryngeal nerve was identified and preserved. Traction was applied to the thyroid gland in a cephalad direction, and the cervical component was completely freed after dividing the inferior thyroid veins with no connection to the intrathoracic goiter (Figure 4). It then became clear that the mediastinal goiter was an ectopic component. The second stage of surgery was performed by the cardiothoracic surgeon via VATS with the right lung collapsed and one‐lung ventilation performed. The right recurrent laryngeal and phrenic nerve were visualized and preserved. The ectopic component was mobilized and completely excised (Figure 5) taking care not to injure the superior lobar pulmonary artery. Patient recovery was uneventful, and she was discharged on postoperative day 3. Final histology of the specimens retrieved showed multinodular goiter for both the right hemithyroidectomy and intrathoracic ectopic thyroid components.

## 6 DISCUSSION

A retrosternal goiter (RSG) is an extension of an enlarged thyroid gland into the mediastinum first described by Haller in 1749. Since then, the terms substernal, intrathoracic, and mediastinal goiters have been used interchangeably, representing the myriad of nomenclatures that may cause confusion and lack of standardization to describe this condition.^4^ It is a relatively common finding, occurring in 1%–20% of all thyroidectomies performed.^5^ Various clinical and radiological classifications of mediastinal goiter have been proposed but none has yet to be universally validated or adopted.^6^ Huinn et al^1^ proposed a classification based on the relationship of the goiter with the aortic arch and right atrium after performing a systematic review on its complications. In Rios et al^6^ study where he critically appraises these classifications, he found that Katlic's definition^7^ which states that a goiter by which at least 50% is retrosternal, is the most useful for predicting the need of a sternotomy.

In the preoperative workup of a RSG, a complete history and physical examination, thyroid function test, thyroid ultrasound (US), and computer tomographic (CT) scan are required. Thyroid US evaluation is useful for evaluation of thyroid nodules to grade and risk‐stratify any nodules according to Thyroid Imaging Reporting and Data System (TIRADS) classification^8^, ^9^ before performing fine‐needle aspiration cytology (FNAC) for cytological correlation. American Thyroid Association guidelines have recommended that thyroid US be the only imaging modality for most thyroid pathologies, but CT is required for retrosternal extension.^10^ The CT scan of the thorax gives vital information for preoperative planning, which include the continuity of the goiter with its intrathoracic component, the anatomical location of the RSG, the relationship of the RSG with its neighboring structures such as the trachea, esophagus, aortic arch, pulmonary vessels, and pericardium. In addition, the morphology of the RSG in particular having an iceberg shape is also an independent predictor of an extra‐cervical approach.^11^ Coskun et al^5^ postulate that the most critical factor to determine whether a RSG can be removed via a cervical approach would be the presence of a clear plane around the goiter in the mediastinum.

Nevertheless, majority of RSG up to 90% can be completely removed via a transcervical approach,^12^ with extra‐cervical approaches such as sternotomy only required in 7% and thoracotomy 3% of the time.^1^ In general, a cervical approach combined with a sternotomy is favoured for a RSG located in the anterior mediastinum whereas a cervical approach with thoracotomy is favoured for one located in the posterior mediastinum.^13^ Blind maneuvers to attempt to dislocate the mediastinal component of the goiter into the neck via passage of a Foley's catheter into the mediastinum, morcellation of the RSG, and insertion of strong silk sutures into the cervical goiter to apply traction from the neck are discouraged due to risk of uncontrolled hemorrhage and injury to adjacent critical structures in the thoracic inlet. Of note, the VATS approach as performed in this case has also been gaining traction in recent years due to its potential for faster recovery and better cosmesis.^13^

The presence of an ectopic RSG in our patient is a rare entity accounting for only 0.2%–1% of all goiters.^2^ An ectopic RSG is postulated to arise from fragments of the developing thyroid that separate from the main body and pulled into the mediastinum during the descent of the heart and great vessels.^14^ The absolute indication for surgery for RSG would be the presence of compressive symptoms such as dysphagia, dyspnea, and superior vena cavae syndrome. Radioactive iodine ablation is an alternative if there are contraindications to surgery in a symptomatic patient and has been estimated to decrease the size of goiter by 40%.^12^ In patients who are asymptomatic, an individualized management plan should take into consideration patient's age, functional status, comorbidities, and size of goiter. We propose that fit young patients with RSG should be offered surgery as they tend to grow and may cause future compressive symptoms. Furthermore, up to 22.6% of RSG may also contain malignancy^14^. Whether or not there is an increased cancer incidence for RSG compared to cervical goiters still remains inconclusive^15^; however, the difficulty of adequately assessing RSG goiters using US or FNA may present with an unknown risk of thyroid cancer that needs to be made known to the patient during counseling. On the other hand, an elderly patient with significant comorbidities with an incidental asymptomatic RSG should be offered active surveillance.

In summary, the surgeon performing thyroidectomy should be well versed with interpreting the radiological features of a RSG as this provides invaluable information with regard to surgical planning, counseling of patient, and subsequent operative strategy. Management plan should be individualized and tailored according to patient’s age, comorbidities, functional status, and clinical symptoms.

## CONFLICT OF INTEREST

None declared.

## AUTHOR CONTRIBUTIONS

We affirm that all individuals listed as authors agree that they have met the criteria for authorship and agree to the conclusions of the study and that no individual meeting the criteria for authorship has been omitted.

## ETHICAL APPROVAL

Informed consent was obtained from the patient regarding the report of her clinical scenario in an anonymous way.
